# Protecting the sugar coat on anti-tumor T cells to conceal destruction by tumor microenvironmental landmines

**DOI:** 10.1016/j.omton.2026.201202

**Published:** 2026-04-27

**Authors:** Lee Seng Lau, Charles J. Dimitroff

**Affiliations:** 1 Department of Cellular and Molecular Medicine, Herbert Wertheim College of Medicine, Florida International University, Miami, FL, USA

## Main text

Adoptive cellular therapies, highlighted by the burgeoning area of chimeric antigen receptor (CAR) T cell therapy, now offer a promising modality with broad application to both hematological and solid cancers. Anti-CD19 CAR T cell therapy, as an example, is an established treatment option for patients with diffuse large B cell lymphoma (DLBCL). However, despite early successes, therapeutic efficacy is limited due to insufficient *in vivo* persistence and exposure to immunosuppressive signals within the tumor microenvironment (TME). To this end, there are ongoing efforts focused on improving T cell longevity and effector function and/or on enabling them to evade TME factors that may impair T cell anti-tumor function.

Coincident with differentiation of naive T cells into effector T cells, notably CD4^+^ and CD8^+^ anti-tumor T cells, is the remodeling of their cell surface sugars, known as glycans. These differentiation-dependent glycans, best characterized by altered asparagine (N)-linked and serine/threonine (O)-glycan structures, are built through the coordinated and regulated expression of glycosyltransferases and glycosidases. Some of the more common alterations are elevations in the complexity and length of glycan antennae, which contain galactose β1,4 N-acetylglucosamine (LacNAc) repeating subunits. Mechanistically, these ß-galactoside-containing glycans evoke molecular vulnerabilities to host and tumor-intrinsic β-galactoside-binding lectins known as galectins. Galectins are a family of 15 β-galactoside-binding lectins and are critical mediators of immunoevasion in cancer.^1^^,^^2^^,^^3^ Galectin (Ga)-1, -3, and -9, in particular, are often upregulated in cancer tissues and in patients with cancer, wherein they avidly bind anti-tumor T cell glycans to compromise their immune-activating, tumor-killing activities and, in other cases, promote apoptosis. Thus, a putative major obstacle in the delivery of optimal CAR T cell therapy is the concomitant abundance of Gal-1, -3 and -9 binding glycans on CAR T cells and the presence of a tumor-promoting, immunosuppressive TME containing Gal-1, -3, and/or -9.

Guiding recent efforts by our laboratory, we postulated that CAR T cells display signature glycan features that heighten their vulnerability to immunosuppressive Gal-1, -3, and/or -9.^4^ Our glycomic, glycosyltransferase expression, and Gal-binding analyses demonstrated that CAR T cells possessed intrinsic glycan features distinct from their non-transduced activated or 10-day expanded T cell counterparts, which rendered them remarkably susceptible to Gal-1 and -3-mediated immunoregulation.^4^ CAR T cell glycans possessed high levels of poly-LacNAc structures on multi-antennary N-glycans, with a conspicuous diminution of β-galactoside α2,6 sialyltransferase 1 (*ST6GAL1*) and corresponding α2,6 sialylated glycans. ST6GAL1 enzymatic activity results in the capping of LacNAc moieties with α2,6-linked sialic acid, truncation of N-glycan antennae, lowering of LacNAc levels, and blocking of Gal-1 and -3-binding.^5^ As expected, CAR T cells bound Gal-1 and -3 significantly more efficiently than their non-transduced activated/expanded T cell counterparts,^4^ due to the marked reduction of ST6GAL1/α2,6 sialylation. Furthermore, clinical data from public mining sources and ELISAs on patients with DLBCL sera galectin levels showed that Gal-3 was highly enriched in lymphoma-associated TMEs, namely in TME macrophages.

Based on these signature CAR T cell glycome and glyco-biosynthetic landscapes, we enforced *ST6GAL1* expression in anti-CD19 CAR T cells (ST6 CAR T cells) to directly examine whether *ST6GAL1* expression in CAR T cells can circumvent Gal-1- and Gal-3-binding and related downstream functional consequences. Our lectin binding and functional assays showed that *ST6GAL1* expression and cell surface α2,6 sialyation inhibited Gal-1- and -3-binding, while avoiding not only Gal-3-dependent pro-apoptotic activity but also improving tumoricidal activity in the presence of Gal-3.^4^ Moreover, cytokine proteome profiling showed that ST6 CAR T cells were spared from Gal-3-dependent production of the Th2 cytokine IL-5 compared with CAR T cell controls. This IL-5-induction effect of Gal-3 binding to CAR T cells was unexpected and, interestingly, markedly compromised tumoricidal activity,^4^ highlighting IL-5 as a potential TME immunosuppressive factor triggered by Gal-3-binding to further impair CAR T cell efficacy.^6^ In pre-clinical anti-tumor assays, we monitored the growth and dissemination of CD19^+^ Raji-luc^+^ lymphoma cells grafted into non-obese diabetic-severe combined immune deficiency (NOD-SCID IL-2Rγ^−/−^) mice treated with ST6 CAR T cells, CAR T cells, or 10-day expanded T cell controls. Bioluminescence intensity and survival data showed significant reductions in tumor growth and increased overall survival in mice treated with ST6 CAR T cells compared with mice treated with CAR T cells. Moreover, there were significantly higher frequencies of ST6 CAR T cells in the spleens compared with splenic numbers of control CAR T cells, implying that *ST6GAL1*/α2,6 sialylation expression enhanced both anti-tumor activity and *in vivo* persistence. Further studies are ongoing to directly investigate the immunocompromising effects of Gal-1 and Gal-3 (or lack thereof) on ST6 CAR T cell treatments in other syngeneic and human tumor xenograft mouse models. Notably, these observations raise the possibility that glycosylation-dependent remodeling may extend beyond immune evasion to influence CAR T cell fitness and metabolic programming. Ongoing studies are focused on defining how altered glycan biosynthesis contributes to cellular persistence and function within the TME.

While a variety of approaches are currently being explored to augment CAR T cell efficacy, including optimization of T cell activation and effector anti-tumor functions via next-generation and combinatorial CAR constructs, glycoengineering is emerging as an exciting complementary strategy to boost anti-tumor immune cell activities. Other recent studies demonstrate that exo-α1,3 fucosylation of antigen-specific T cells or CAR T cells can improve tumor-infiltrating capacity and anti-tumor activity,^7^ whereas enforcing *ST6GAL1*/α2,6 sialylation expression on natural killer (NK) cells enhances NK cell-mediated killing of CD22^high^ B cell malignancies through improved Siglec-2 (CD22) interactions.^8^ Indeed, other glycoengineering strategies, besides enforced glycosyltransferase gene expression or exo-glycosylation of the glycocalyx, such as glycosyltransferase gene silencing, metabolic inhibition of glycosyltransferases/glycosidases,^9^ or the use of specific small-molecule inhibitors of Gal-1, -3 and/or -9, offer promising avenues for improving CAR T cell efficacy, particularly for use against malignancies characterized by high levels of Gal-1 and/or -3 in their TME.

**Figure 1:**
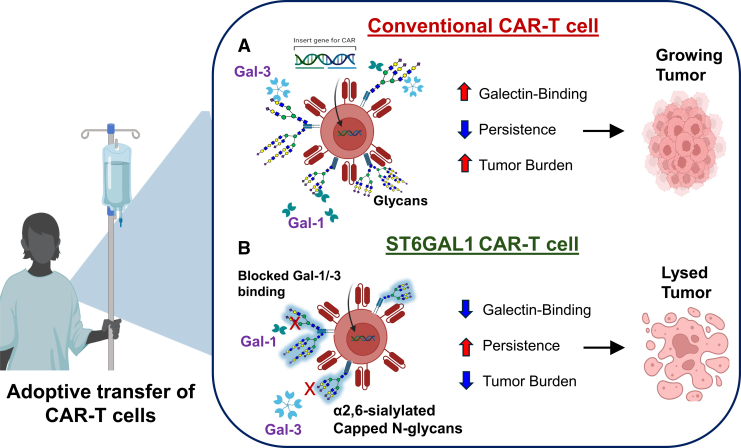
Enforcing α2,6 sialylation on CAR-T cells protects them from immunosuppressive Gal-1 and Gal-3 (A) A cartoon model depicting the impact of Gal-1 and -3 on conventional anti-tumor CAR T cell function and (B) the protective role of enforced *ST6GAL1*/α2,6 sialylation on Gal-1 and -3-dependent CAR T cell immunoregulation. Created using BioRender illustrating tool (Biorender.com).

In summary, our recent data provide a better understanding of the inherent glyco-molecular vulnerabilities of CAR T cells to Gal-1 and -3 binding and related downstream immunoregulatory activities. While glycan remodeling occurs in T cells undergoing antigen activation and clonal expansion, we find, surprisingly, that other glycome and glycan biosynthetic changes arise during lentiviral transduction and CAR T cell manufacturing processes. Our findings support enforced expression of α2,6 sialylation as a model to protect the “glycan” coat on CAR T cells from binding Gal-1 and -3 and to evade the deleterious consequences on cell viability and deregulated cytokine production, thereby boosting *in vivo* persistence and antitumor efficacy (Figure 1). These findings establish glycoengineering as a novel and clinically relevant strategy to overcome TME-mediated immunosuppression and improve CAR T cell efficacy.

## Acknowledgments

This work was supported by the following grants: NIH/NCI R01CA282520 (CJ Dimitroff) and the FIU/HWCOM-Baptist Health Pilot Grant (CJ Dimitroff). The content is solely the responsibility of the authors and does not necessarily represent the official views of the funding sources.

## Author contributions

Conceptualization, L.S.L. and C.J.D.; writing – original draft, L.S.L.; writing—review and editing, L.S.L. and C.J.D.; funding acquisition, C.J.D.

## Declaration of interests

The authors declare no conflict of interest.
